# Estrogen suppresses adipogenesis by inhibiting S100A16 expression

**DOI:** 10.1530/JME-13-0273

**Published:** 2014-06

**Authors:** Rihua Zhang, Dongming Su, Weidong Zhu, Qiong Huang, Menglan Liu, Yi Xue, Yuanyuan Zhang, Dong li, Allan Zhao, Yun Liu

**Affiliations:** 1 Department of Geratology The First Affiliated Hospital, Nanjing Medical University Nanjing, 210029 China; 2 Laboratory Animal Center, The First Affiliated Hospital, Nanjing Medical University Nanjing, 210029 China; 3 The Center of Metabolism, Nanjing Medical University Nanjing, 210029 China; 4 Department of Urology Zhongda Hospital Affiliated to Southeast University Nanjing, 210008 China; 5 Department of Orthopedics Jiangsu Province Hospital of TCM Affiliated Hospital of Nanjing University of TCM Nanjing, Jiangsu China; 6 Department of Endocrinology Changzhou Wujin People's Hospital 213000, Changzhou, Jiangsu China

**Keywords:** estrogen, metabolic syndrome, S100A16

## Abstract

The aim of this study is to determine the effects of E_2_ on metabolic syndrome and the molecular mechanisms involving S100A16. Ovariectomized (OVX) rat models and mouse embryonic fibroblasts cell models were used. E_2_ loss in OVX rats induced body weight gain and central abdominal fat accumulation, which were ameliorated by E_2_ treatment under chow and high-fat diet (HFD) conditions. E_2_ decreased the expression of the adipocyte marker genes *PPAR* *γ*, aP2, *C/EBP* *α*, and *S100A16*. E_2_ inhibited adipogenesis. Overexpression of S100A16 reversed the E_2_-induced adipogenesis effect. A luciferase assay showed that E_2_ inhibited the expression of S100A16. E_2_ treatment decreased body weight gain and central abdominal fat accumulation under both chow and HFD conditions. Also, E_2_ suppressed adipogenesis by inhibiting S100A16 expression.

## Introduction

In postmenopausal women, estrogen loss is an independent risk factor for more severe menopausal symptoms, such as metabolic disease, insulin resistance, and type 2 diabetes (Carr 2003, Manrique *et al*. 2012). Studies on mouse models have shown that oophorectomy results in obesity, altered fat distribution, adipose tissue inflammation, and development of fatty liver (Rogers *et al*. 2009, Stubbins *et al*. 2012). Estrogen therapy is believed to have beneficial effects on abdominal fat accumulation, insulin resistance, and development of type 2 diabetes in postmenopausal women (Sørensen *et al*. 2001, Stubbins *et al*. 2012). However, there are reports that estrogen treatment does not improve insulin action in humans and rodents (Basu *et al*. 2007); furthermore, it is associated with a higher risk of insulin resistance and type 2 diabetes in postmenopausal women (Ryan *et al*. 2002, Ding *et al*. 2007). The longitudinal data from the Study of Women's Health Across the Nation (SWAN), which included five ethnic groups in the USA – African-Americans, Caucasians, Chinese, Hispanic, and Japanese – indicated that early use of hormone therapy was a risk factor for obesity (Sutton-Tyrrell *et al*. 2010), and there was little evidence to suggest that estrogen application was beneficial for insulin resistance and stroke (Hu & Grodstein 2002, Golden *et al*. 2007). Therefore, the relationship between estrogen use and metabolic disorders in postmenopausal women remains controversial. Moreover, the therapeutic effects of estrogen may be influenced by many factors, such as the dosage, the duration of treatment, and the type of estrogen used. Another disadvantage of the clinical application of estrogen is its reported growth-promoting effects on the uterus or mammary gland and tumor-promoting actions (Królik & Milnerowicz 2012, Nichols *et al*. 2012). Further studies are therefore needed to re-evaluate the benefits of estrogen treatment and to determine the estrogen dose at which the risk of metabolic diseases is increased.

The mechanisms via which estrogen affects metabolism are complicated and not well known. It is reported that the anti-obesity effect of estrogen depends on leptin and Stat3 activation (Gao *et al*. 2007). In our previous study (Liu *et al*. 2011), we found that a novel adipogenesis-promoting factor, the S100A16 protein, which is a member of the S100 protein family, plays a role in the action of estrogen. We analyzed the *S100A16* promoter using AliBaba2 (Grabe 2002), and found four estrogen response elements (EREs) within the 1500-bp promoter region. Therefore, we think that estrogen may regulate lipid metabolism by mediating S100A16 expression.

In this study, we used normal rats and ovariectomized (OVX) rats that received estrogen treatment or control treatment under chow and high-fat diet (HFD) conditions. We wanted to determine which physiological indicators of metabolic disease benefit from estrogen administration, and evaluate the effect of estrogen on *S100A16* expression.

## Materials and methods

### Animals

Fifty healthy female specific pathogen-free Sprague–Dawley (SD) rats 6 weeks of age (body weight, 150–200 g) were purchased from and housed at the Experimental Animal Center of Nanjing Medical University (Nanjing, China). The experiments were approved by the Nanjing Medical University Ethical Committee. Animals were housed at 23±1 °C with a 12 h light:12 h darkness cycle and 45±5% humidity and allowed free access to normal chow diet and water. Thirty-two female SD rats underwent bilateral oophorectomy at the age of 8 weeks under general anesthesia induced with ketamine (120 mg/kg, i.p.). After 2 weeks of recovery, the rats were allowed free access to water and were fed normal powder chow or HFD. The HFD was supplied by OpenSource DIETS (Research Diets, Inc., New Brunswick, NJ, USA, #D12451). Sixteen OVX rats received s.c. injections (at the back of the neck) of 200 μg/kg 17β-estradiol (Sigma, #E2758) twice a week for 16 weeks. Control rats (*n*=16) received PBS injections. We measured body weight every week after the first injection.

Three groups of rats were on the chow diet: normal control rats (*n*=9); OVX rats (*n*=8); and OVX rats given E_2_ treatment (*n*=8). There were also three groups of rats on HFD: normal control rats (*n*=9); OVX rats (*n*=8); and OVX rats given E_2_ treatment (*n*=8). All procedures were approved by the Experimental Animal Center of Nanjing Medical University.

To assess the effects of E_2_ levels on body weight gain, we monitored the body weight of all rats every week and measured visceral fat weight when the rats were anesthetized with Nembutal (100 mg/kg). All protocols involving the use of animals have been approved by the Institutional Animal Care and Use Committee (IACUC) at the University of Nanjing Medical University. In addition, we investigated the morphology of visceral fat cells from all rats fed normal chow and HFD by using hematoxylin and eosin staining.

### Intraperitoneal glucose tolerance test

Fourteen weeks after the E_2_ treatment, IPGTT was conducted. All rats were starved for 12 h, and then their tail blood glucose concentrations (mM) were monitored using a handheld glucometer (ACCU-CHEK Performa, Roche). Blood was collected from the inner canthus to detect insulin concentration. Next, all rats received i.p. injections of glucose (2 g/kg body weight); tail blood glucose concentrations were measured using the handheld glucometer and blood was collected from the inner canthus at 15, 30, 60, and 120 min. Blood samples were analyzed for insulin at every time point using an immunoradiometric assay kit (#KAP1251) supplied by DIAsource ImmounoAssays S.A (Louvain-La-Neuve, Belgium).

### Measurement of E_2_ and liver and kidney function indicators

At the end of 16 weeks of E_2_ treatment, all rats were anesthetized with ketamine (120 mg/kg i.p.), and blood was collected by cardiac puncture from the left ventricle into tubes precoated with potassium-EDTA, and centrifuged at 3000 ***g*** and 4 °C for plasma preparation. The plasma concentrations of E_2_ were quantified using immunoradiometric assay kits supplied by DIAsource ImmounoAssays S.A. (#KIP0629). The plasma levels of total cholesterol (TC), triglyceride (TG), LDL, HDL, alanine transaminase (ALT), lactate dehydrogenase (LDH), creatinine (Cr), urea, and uric acid (UA) were determined by the Medical Laboratory of Jiangsu Province Hospital, The First Affiliated Hospital of Nanjing Medical University. All assays were performed according to the manufacturer's instructions.

Visceral fat was removed rapidly; part of the samples was frozen in liquid N_2_ and stored at −80 °C for the extraction of protein, and part of the samples was formalin fixed and paraffin embedded for the production of pathological sections. The same procedure was performed on the rest of the tissues.

### Mouse embryonic fibroblast isolation and differentiation

Mouse embryonic fibroblasts (MEFs) were isolated from the embryos of C57BL/6 and S100A16^Tg^^+^^/^^+^ mice at 13.5 days post coitum. The construct was generated by inserting *S100A16* cDNA into a vector with the *PCAG* promoter. We then obtained transgenic mice by the microinjection method. This F0 transgenic mouse was bred with the C57BL/6 mouse to obtain F1 transgenic mouse lines. *S100A16*-positive transgenic mice and their genotype were confirmed by PCR, and the expression of *S100A16* was determined by qPCR and western blot. Embryos were chopped and incubated in a 0.25% trypsin and 0.01% EDTA solution at 37 °C for 10 min. The cells dispersed by pipetting were washed with PBS and then maintained in DMEM (Gibco) containing 10% fetal bovine serum (FBS) (Hyclone, Thermo Scientific, Logan, UT, USA), 100 U/ml penicillin, and 0.1 mg/ml streptomycin in a 5% CO_2_ atmosphere at 37 °C. To induce adipocyte differentiation, 2-day post-confluent MEFs (designated day 0) were cultured with DMEM containing 10% FBS and MIX (0.5 mmol/l 3-isobutyl-1-methyxanthine, 1 μg/ml porcine insulin, and 1 mmol/l dexamethasone (Sigma)). After 48 h of incubation (designated day 2), the medium was replaced with DMEM containing 10% FBS and 1 μg/ml insulin.

### Oil Red O staining

Differentiated MEFs (day 10) were washed three times with PBS and stained with filtered Oil Red O solution (stock solution, 3 mg/ml in isopropanol; working solution, 60% of the stock solution and 40% distilled water) for 30 min at room temperature. The cells were then washed with ddH_2_O to remove unbound dye, visualized, and photographed under a microscope.

### TG GPO-POD assay

Cellular TG content was determined using a TG GPO-POD assay kit (Sigma). MEFs were cultured and induced in a 10-cm well by MIX to differentiate into adipocytes (10 days), washed with PBS twice, scraped into 500 μl PBS, sonicated to homogenize the suspension, and then assayed for total TG content.

### Protein extraction and western blotting

Tissues and cells were washed twice with ice-cold PBS. Then, 100 mg tissue was lysed with 1 ml lysis buffer (50 mM Tris–HCl (pH 7.4), 150 mM NaCl, 1% (v/v) Nonidet-P40, 1 mM EDTA, 1 mM NaF, 10 μg/ml aprotinin, 10 μM leupeptin, and 1 mM phenylmethanesulfonyl fluoride). The cells were scraped into the lysis buffer, and then tissues and cells were allowed to stand on ice for 30 min. After centrifugation at 4 °C, the proteins in the supernatant were extracted and separated on SDS–polyacrylamide gels before being subjected to a standard western blot assay and imaged using Molecular Imager ChemiDoc XRS+ with the Image Lab Software (Version 4.0.1, Bio-Rad Laboratories, Hercules, CA, USA).

### Luciferase assay

**Luciferase reporter plasmid construction.** The *S100A16* promoter (1500 bp) was amplified by PCR using primers terminating in an *XhoI* and *HindIII* recognition sequence. The PCR product was digested and ligated into a pGL3-basic vector.

**Transient transfection and luciferase assay.** The day before transfection, CHO cells were plated on 12-well cell culture plates at 3×10^5^ cells/well. Transfection was performed using the X-tremeGENE HP DNA Transfection Reagent (#06365752001, Roche), following the manufacturer's protocol. Six hours later, cells were treated with E_2_ (400 pg/ml) and/or tamoxifen (0.05 μM) for 24 h. The Renilla luciferase reporter plasmid pRL-SV40 was used as an internal control. Luciferase activity was measured using the dual luciferase assay system (Promega, E1910) with a luminometer (GloMax 20/20 Luminometer, Promega (Turner)). Luciferase assays were performed in triplicate and repeated at least three times to confirm their reproducibility.

### Statistical analyses

The *in vitro* and *in vivo* results were analyzed using one-way ANOVA. *P*<0.05 was considered to indicate statistical significance. The results are presented as mean±s.d. of the values from three to six replicates per group.

## Results

### The effect of E_2_ on body weight gain, visceral fat weight, and biochemical traits under both the chow and HFD conditions

We measured E_2_ concentrations at the time the animals were killed. Under the chow conditions, in comparison to the control rats, E_2_ concentrations increased in the OVX rats treated with E_2_ (759.11±53.22 vs 77.09±5.21 pg/ml) and decreased in the OVX rats not treated with E_2_ (42.0±3.02 vs 77.09±5.21 pg/ml); also under the HFD conditions, E_2_ concentrations increased in the OVX rats treated with E_2_ (1209.08±198.02 vs 100.82±14.09 pg/ml) and decreased in the OVX rats not treated with E_2_ (34.0±3.15 vs 100.82±14.09 pg/ml) (Table 1).

Under the chow and HFD conditions, the high level of E_2_ was more efficacious at lowering the percentage of body weight gain and visceral fat weight than control treatment. Under both diet conditions, low levels of E_2_ had the opposite effect. Under the chow conditions, the percentage of body weight gain was 5.32% in the high-E_2_ group, 61.40% in the low-E_2_ group, and 40.62% in the control group. Visceral fat weight was 4.7±0.91 g in the high-E_2_ group, 35.1±1.83 g in the low-E_2_ group, and 20.8±1.75 g in the control group (Table 1). Under the HFD conditions, the percentage of body weight gain was 8.25% in the high-E_2_ group, 64.71% in the low-E_2_ group, and 38.23% in the control group. Visceral fat weight was 12.06±1.04 g in the high-E_2_ group, 42.8±2.14 g in the low-E_2_ group, and 31.3±2.06 g in the control group (Table 1).

**Figure 1:**
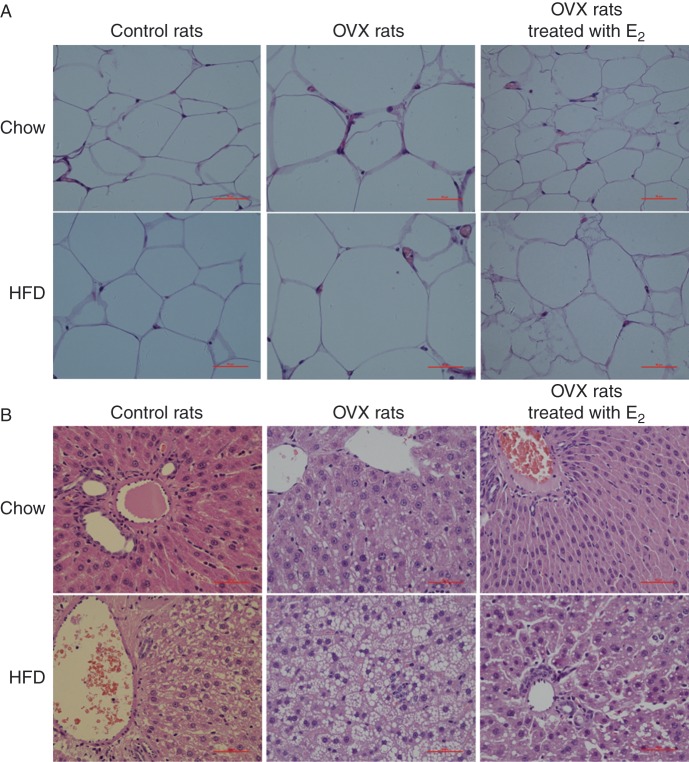
Images of visceral fat cells and liver cells. (A) Images of visceral fat cells after hematoxylin and eosin (HE) staining. The body weights of all rats were monitored every week and visceral fat weight was measured when the mice were anesthetized with Nembutal (100 mg/kg). The images are shown at 400× magnification. (B) Histological images of the livers of rats fed chow and HFD, as visualized by HE staining. All the images are shown at 400× magnification.

Under both diet conditions, the size of visceral fat cells was decreased in the E_2_-treated OVX rats, and it was increased in the OVX rats. These results were similar to the body weight changes and confirm that E_2_ inhibited body weight gain (Fig. 1A).

The plasma levels of TC and TGs increased in OVX rats, and returned to normal levels with E_2_ treatment under both conditions (Table 2). There were no changes in HDL and LDL levels under these conditions. which was decreased using E_2_ treatment in the two conditions (Table 2).

The levels of ALT and LDH, which are common biomarkers for liver damage, were determined in plasma samples. Under both conditions, the level of ALT and LDH were increased, which were reduced by E_2_ treatment (Table 3). Morphological analysis of liver cells showed that compared with chow-fed rats, the livers of rats fed HFD appeared to be filled with macrovesicular fat within the hepatocytes. Importantly, E_2_ treatment reduced lipid accumulation in the liver under both conditions (Fig. 1B).

The plasma levels of UA in the OVX group was increased but the difference was not significant. Whilst the levels for the E_2_-treated OVX group were lower than those for the OVX group, in the HFD group the levels were higher than in the control group and in the Chow group the levels were lower. Both these differences were significant, implying that the E_2_-treated groups differed from the control group in opposite directions (Table 3).

### The effect of E_2_ on systemic glucose homeostasis

**Figure 2:**
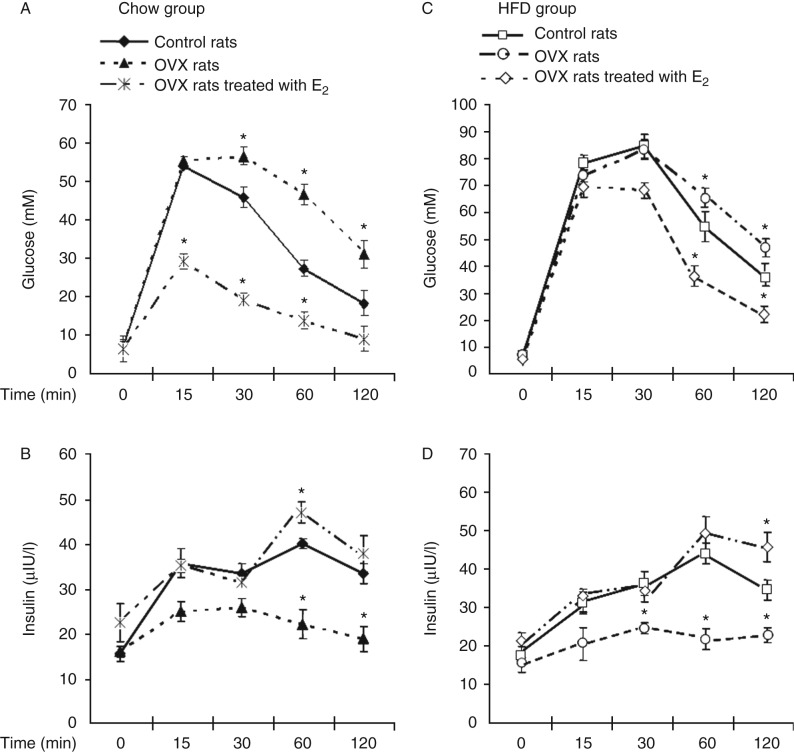
The effect of E_2_ on systemic glucose homeostasis. (A) Glucose level of rats under the chow conditions. (B) Insulin level of rats under the chow conditions. (C) Glucose level of rats under the HFD conditions. (D) Insulin level of rats under the HFD conditions. Results are expressed as the mean±s.d. *P<0.05 compared with the corresponding control group.

The effect of E_2_ on systemic glucose homeostasis was evaluated by the IPGTT. Under the chow conditions, blood glucose reached its maximum at 15 min in all rats. Interestingly, the maximum blood glucose level was 53.3±4.3 and 54.7±3.2 mM in the normal and OVX rats, respectively, but only 28.4±2.3 mM in the OVX rats treated with E_2_. Subsequently, the blood glucose level gradually decreased in all rats. Importantly, the blood glucose of control and OVX rats treated with E_2_ dropped to low levels at 120 min, but the blood glucose of the OVX rats not treated with E_2_ was higher than physiological concentrations at 120 min (32.2±2.2 vs 6.1±1.1 mM; Fig. 2A). The OVX rats had already become glucose-intolerant at this time. When compared with control rats, glucose-stimulated insulin secretion was abolished and insulin concentration remained low in the OVX rats but not E_2_-treated OVX rats (Fig. 2B).

Under the HFD conditions, blood glucose reached its maximum at 30 min in normal and OVX rats, at 88.6±7.7 and 87.2±6.5 nM, respectively, but the maximum was 71.1±4.3 nM at 15 min in the OVX rats treated with E_2_. The blood glucose of the normal and the OVX rats decreased but did not drop to normal levels (42.2±3.1 vs 6.1±1.1 nM). The blood glucose of the OVX rats treated with E_2_ dropped to close to the normal levels, at 20.4±5.7 nM at 120 min (Fig. 2C). Glucose-stimulated insulin secretion was abolished and insulin concentration remained low in the OVX rats but not E_2_-treated OVX rats (Fig. 2D). The OVX rats had already become glucose-intolerant at this time.

### The effect of E_2_ on the expression of adipogenesis marker genes *PPAR* *γ*, *C/EBP* *α*, and aP2 in fat tissues

**Figure 3:**
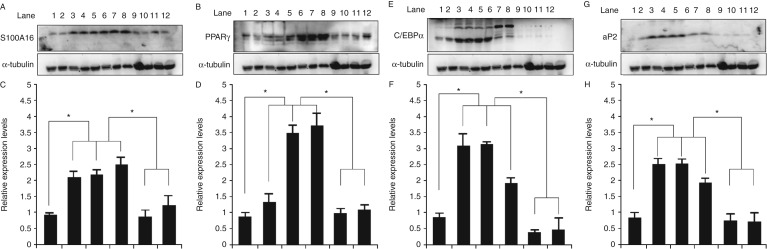
The effect of E_2_ on the expression of adipogenesis marker genes of *PPAR* *γ*, *C/EBP* *α*, and aP2 in fat tissue. The protein expression of S100A16 (A), PPARγ (B), C/EBPα (E), and aP2 (G) was analyzed with western blotting. (C, D, F and H) Relative expression of *S100A16*, *PPAR* *γ*, *C/EBP* *α*, and aP2 based on grayscale analysis. α-Tubulin was used as the control. Lanes 1 and 2, normal control rats on the chow diet; lanes 3 and 4, normal control rats on the HFD; lanes 5 and 6, OVX rats on the chow diet; lanes 7 and 8, OVX rats on the HFD; lanes 9 and 10, OVX rats treated with E_2_ on the chow diet; lanes 11 and 12, OVX rats treated with E_2_ on the HFD. Results are expressed as the mean±s.d. *P<0.05 compared with the corresponding control group.

Differentiation of preadipocytes to adipocytes requires coordinated action of a large repertoire of transcription factors, such as *PPAR* *γ*, *C/EBP* *α*, and aP2. *S100A16* is a novel adipogenesis-promoting factor. In order to study the mechanism of E_2_-suppression of body weight gain, we detected the expression of these proteins in the fat tissue of all groups. According to the results of western blotting, the expression of *S100A16*, *PPAR* *γ*, *C/EBP* *α*, and aP2 was significantly elevated in fat tissues from non-treated OVX rats fed chow or HFD. In sharp contrast to these observations, these proteins only showed a slight increase in the fat tissues from OVX rats treated with E_2_ under both conditions (Fig. 3A, B, C, D, E, F, G and H).

### The role of S100A16 in E_2_-induced suppression of adipogenesis

**Figure 4:**
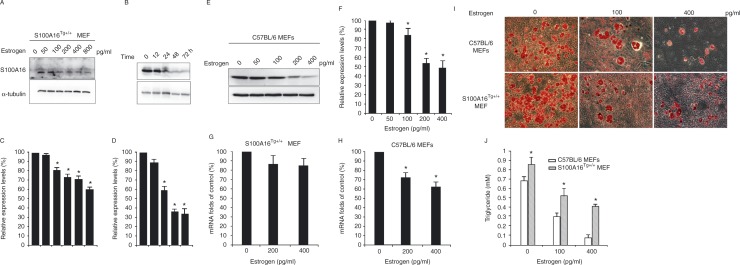
E_2_-induced suppression of adipogenesis via S100A16 inhibition. (A) Western blot assays of S100A16 expression in the protein extracts collected from S100A16^Tg^^+^^/^^+^ MEFs treated with different concentrations of E_2_ (0, 50, 100, 200, 400, and 800 pg/ml) for 24 h. (B) Western blot assays of S100A16 expression in the protein extracts collected from S100A16^Tg^^+^^/^^+^ MEFs treated with 100 pg/ml E_2_ for different times (0, 12, 24, 48, and 72 h). α-Tubulin was used as the control. (E) Western blot assays of S100A16 expression in the protein extracts collected from C57BL/6 MEFs treated with different concentrations of E_2_ (0, 50, 100, 200, and 400 pg/ml) for 24 h. (C, D and F) Relative expression of S100A16 based on grayscale analysis. (G and H) mRNA levels of *S100A16* S100A16^Tg^^+^^/^^+^ MEFs and normal MEFs, respectively, treated with E_2_. (I) Oil Red O staining patterns for MEFs from C57BL/6 and S100A16^Tg^^+^^/^^+^ mice treated with different concentrations of E_2_ and induced to differentiate into adipocytes. Photographs were taken under a light microscope with 200× magnification. (J) Triglyceride accumulation in the MEFs was significantly higher in the S100A16^Tg^^+^^/^^+^ MEFs for all E_2_ concentrations. *P<0.05 compared with the corresponding control group.

When compared with control cells, E_2_-treated S100A16^Tg^^+^^/^^+^MEFs (preadipocytes) showed a significant decrease in S100A16 expression in a dose-dependent manner (Fig. 4A and C). Stimulation of S100A16^Tg^^+^^/^^+^ MEFs with 100 pg/ml E_2_ (Fig. 4B and D) showed a significant change in S100A16 protein expression after 24 h. Therefore, E_2_ inhibited S100A16 expression in a dose- and time-dependent manner. We also detected the effect of E_2_ on endogenous S100A16 expression using normal MEFs from C57BL/6 mice. The results indicated that E_2_ inhibited S100A16 expression (Fig. 4E and F). We examined the effect of E_2_ on the levels of *S100A16* mRNA in normal MEFs and S100A16^Tg^^+^^/^^+^ MEFs. The results showed that E_2_ suppressed the *S100A16* mRNA expression in normal MEFs, but had no effect on the *S100A16* mRNA expression in S100A16^Tg^^+^^/^^+^ MEFs (Fig. 4G and H).

To confirm whether E_2_ suppressed adipogenesis by inhibiting the expression of S100A16, MEFs isolated from S100A16^Tg^^+^^/^^+^ and C57BL/6 mice were treated with different concentrations of E_2_ and stained with Oil Red O. E_2_ treatment resulted in sparse staining, which was ameliorated by the overexpression of S100A16 (Fig. 4I). Consistent with the staining patterns, quantitative analysis of cellular TGs showed that TG accumulation was significantly lower in the C57BL/6 MEFs treated with E_2_ (400 pg/ml) than the S100A16^Tg^^+^^/^^+^ cells (Fig. 4J). The results indicate that overexpression of S100A16 reversed reduction of TG accumulation induced by E_2_.

### Effect of E_2_ on S100A16 transcription

**Figure 5:**
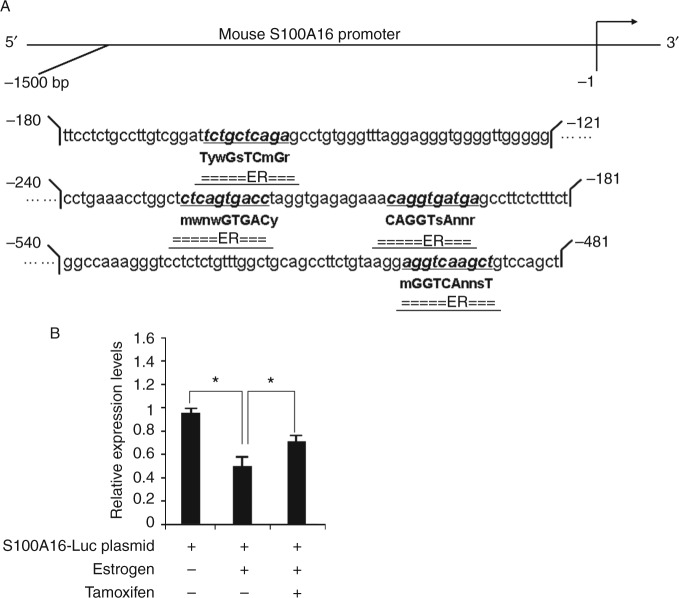
Inhibition of S100A16 transcription by E_2_. (A) The 1500-bp promoter region of *S100A16* shows four estrogen receptor (ER) binding sites. (B) Results of the S100A16 luciferase reporter assay; S100A16 expression was reduced in the presence of E_2_, and this effect was ameliorated by the addition of tamoxifen. Results are expressed as mean±s.d. *indicates statistical significance (*P*<0.05 ).

We predicted the transcription factors that may bind to the promoter region of *S100A16*, using bioinformatics (http://www.cbrc.jp/htbin/nph-tfsearch). The *S100A16* promoter has numerous transcription factor-binding sites, and also four estrogen receptor (ER)-binding sites (Fig. 5A).

The 1.44-kb upstream region of mouse S100A16 was constructed into a luciferase reporter (pGL3-basic). In CHO cells, E_2_ reduced the transactivation of this reporter, which was partly reversed by tamoxifen (an inhibitor of ER; Fig. 5B). The data indicated the possibility that E_2_ inhibited S100A16 expression, at least in part, by acting as a negative transcriptional regulator of *S100A16*.

## Discussion

In the present study, we studied the effect of different estrogen dosages on weight gain, insulin sensitivity, glucose and lipid metabolism, differentiation of MEFs, and liver and renal function in OVX and control female rats fed chow and HFD.

Our data indicated that estrogen depletion induced body weight gain and central abdominal fat accumulation, which were ameliorated by estrogen therapy under both diet conditions (Table 1). We also found that E_2_ loss induced glucose metabolism disorder and insulin resistance under different diet conditions (Fig. 2), and subsequently led to the development of metabolic syndrome. This is in agreement with results of studies which showed that E_2_ loss was positively associated with insulin resistance (Kalish *et al*. 2003). The features of the metabolic syndrome are related to the accumulation of visceral adiposity, insulin resistance, and disorder of biomarkers of liver and kidney. Although high amounts of abdominal fat are thought to promote insulin resistance (Ryan 2000, Soares *et al*. 2006), in our study, E_2_ treatment inhibited body weight and visceral fat gain, and alleviate insulin resistance. With regard to diet, we found that E_2_ improved glucose-stimulated insulin secretion in OVX rats under the chow and HFD conditions, and that it was beneficial for maintaining blood glucose homeostasis (Fig. 2). Moreover, E_2_ normalized the levels of TC, TG, and UA that had been increased in the OVX rats, under the chow and the HFD conditions. HDL and Cr displayed no changes, while LDH and ALT displayed no changes in the OVX rats but decreased in E_2_-treated rats (Tables 2 and 3). E_2_ treatment reduced lipid accumulation in the liver under both conditions (Fig. 1B). UA is an independent risk factor for metabolic syndrome. Our results showed that E_2_ loss in OVX rats obviously increase the level of UA whether under the chow conditions or under the HFD conditions, but E_2_ replacement therapy could reverse this phenomenon (Table 3).

Little is known about the molecular mechanism underlying E_2_-induced weight loss. This complex process is regulated by many cell signals. For example, E_2_ binding to ERs (ERα and ERβ) resulted in the initiation of signal transduction. E_2_ may exert the effect of inhibiting body weight gain via leptin-like effects (Gao *et al*. 2007). In our previous study, we first found a new gene, *S100A16,* related to obesity, which is a member of the S100 protein family (Liu *et al*. 2011). It is interesting that we found four EREs in the promoter of *S100A16* within the 1500 bp by using AliBaba2 analysis (Fig. 5A). Therefore, we speculated that estrogen might regulate metabolism progress by mediating S100A16 expression. To verify this hypothesis, we examined the expression of S100A16 as well as the adipocyte marker genes *PPAR* *γ*, *C/EBP* *α*, and aP2 in the fat tissues from the different groups of rats mentioned earlier. The results showed that E_2_ treatment inhibited the expression of adipocyte marker genes, including *PPAR* *γ*, *C/EBP* *α*, and aP2, and of *S100A16* (Fig. 3), which was consistent with the previous studies (Alonso-Vale *et al*. 2009, Yim *et al*. 2011). To further determine whether E_2_ acts via the regulation of S100A16 expression, MEFs were extracted from the S100A16^Tg^^+^^/^^+^ mice bred in our laboratory and incubated with E_2_ at different dosages and for different times, we found that the E_2_ inhibited S100A16 expression in a dose- and time-dependent manner (Fig. 4A, B, C and D). At the same time, we observed the role of E_2_ in the differentiation of MEFs extracted from S100A16^Tg^^+^^/^^+^ mice and control C57BL/6 mice by Oil Red O staining and TG determination. It was significant that S100A16 promoted the differentiation of MEFs and that E_2_ inhibited the effects of S100A16 (Fig. 4E and F). Results of the S100A16 luciferase reporter assay also showed that S100A16 expression was reduced in the presence of E_2_, and this effect was ameliorated by the addition of tamoxifen (Fig. 5B).

So we think that estrogen might regulate metabolism by mediating S100A16 expression. Though many studies must be processed to reveal the molecular mechanisms of the effects of estrogen on metabolism via the S100A16 pathway. Indeed, our data showed that E_2_ inhibited adipogenesis and S100A16 expression, which indicates that E_2_ might decrease S100A16 expression by inhibiting *S100A16* gene transcription. Our study has revealed the interaction of S100A16 with estrogen, and will be applicable to clinical treatment involving estrogen.

## Author contribution statement

R Z, D S, and W Z conducted the molecular and the animal studies, carried out the data collection, and wrote this paper; Y X, D L, M L, Q H, and Y Z conducted the animal studies; A Z conducted part of the molecular studies; and Y L designed this study.

## Figures and Tables

**Table 1:** E_2_ inhibited body weight gain and visceral fat weight increase under both the chow and the HFD conditions. Plasma concentration of E_2_, body weight gain, and visceral fat weight in the different groups of rats. Results are expressed as mean±s.d.

**Diet**	**Plasma E_2_** (pg/ml)			**Body weight gain** (%)			**Visceral fat weight** (g)		
Normal	OVX	OVX+E_2_	Normal	OVX	OVX+E_2_	Normal	OVX	OVX+E_2_
Chow	77.09±5.21	42±3.02*	759.11±53.22*	40.62±5.27	61.40±5.33*	5.32±1.01*	20.8±1.75	35.1±1.83*	4.7±0.91*
HFD	100.82±14.09	34±3.15*	1209.08±198.02*	38.23±5.03	64.71±6.24*	8.25±0.15*	31.3±2.06	42.8±2.14*	12.06±1.04*

*P<0.05 compared with the corresponding control group. OVX, ovariectomized; HFD, high-fat diet; E_2_, estrogen.

**Table 2:** The effect of E_2_ on TC, TG, LDL, and HDL under both the chow and the HFD conditions. Plasma level of TC, TGs, LDL, and HDL in the different groups of rats. Results are expressed as mean±s.d.

**Diet**	**TC** (mmol/l)			**TG** (mmol/l)			**LDL** (mmol/l)			**HDL** (mmol/l)		
Normal	OVX	OVX+E_2_	Normal	OVX	OVX+E_2_	Normal	OVX	OVX+E_2_	Normal	OVX	OVX+E_2_
Chow	2.16±0.16	2.84±0.09*	2.18±1.12*	0.67±0.26	1.01±0.18*	0.56±0.1*	0.23±0.14	0.39±0.08	0.19±0.07	1.43±0.14	1.67±0.08	1.5±0.07
HFD	2.23±0.27	3.3±0.15*	2.32±0.29*	1.11±0.08	1.59±0.18*	1.09±0.11*	0.17±0.09	0.29±0.08	0.17±0.12	1.44±0.09	1.41±0.08	1.36±0.12

*P<0.05 compared with the corresponding control group. TC, total cholesterol; TGs, triglycerides; OVX, ovariectomized; HFD, high-fat diet; E_2_, estrogen.

**Table 3:** The effect of E_2_ on ALT, LDH, Cr, and UA under both the chow and the HFD conditions. Plasma level of total ALT, LDH, Cr, and UA in the different groups of rats. Results are expressed as mean±s.d.

**Diet**	**ALT** (U/l)			**LDH** (U/l)			**Cr** (μm/l)			**Plasma UA** (μm/l)		
Normal	OVX	OVX+E_2_	Normal	OVX	OVX+E_2_	Normal	OVX	OVX+E_2_	Normal	OVX	OVX+E_2_
Chow	64.14±3.24	66.26±5.38	47.04±5.07*	1171.4±0.14	1185.86±0.08	941.29±0.07*	45.7±0.14	47.21±0.08	49.47±0.07	44.86±0.26	131.91±0.18	37.96±0.1*
HFD	57.74±4.29	53.55±4.07	40.77±7.22*	1179.71±0.09	1602.83±0.08	790.19±0.12*	45.06±0.09	49.72±0.08	48.57±0.12	59.51±0.08	91.07±0.18	61.02±0.11*

*P<0.05 compared with the corresponding control group. ALT, alanine transaminase; LDH, lactate dehydrogenase; Cr, creatinine; UA, uric acid; OVX, ovariectomized; HFD, high-fat diet; E_2_, estrogen.
